# Photocatalytic TMO-NMs adsorbent: Temperature-Time dependent Safranine degradation, sorption study validated under optimized effective equilibrium models parameter with standardized statistical analysis

**DOI:** 10.1038/srep42509

**Published:** 2017-02-14

**Authors:** Rizwan Wahab, Farheen Khan, Nagendra Kumar Kaushik, Javed Musarrat, Abdulaziz A. Al-Khedhairy

**Affiliations:** 1 Zoology Department, College of Science King Saud University, Riyadh 11451, Saudi Arabia; 2 Al-Jeraisy, Chair for DNA Research, Department of Zoology, College of Science, King Saud University, Riyadh 11451, Saudi Arabia; 3 Department of Chemistry, Aligarh Muslim University, Aligarh U.P. 202002, India; 4 Plasma Bioscience Research Center, Kwangwoon University, Seoul 139701, South Korea; 5 Dept. of Ag. Microbiology, AMU, Aligarh, India; 6 Baba Gulam Shah Badshah University, Rajouri, J&K, India

## Abstract

In this paper, chemically synthesized copper oxide nanoparticles (CuO-NPs), were employed for two processes: one is photocatalytic degradation and second one adsorption for the sorption of safranine (SA) dye in an aqueous medium at pH = 12.01. The optimized analytes amount (nano-adsorbent = 0.10 g, conc. range of SA dye 56.13 ppm to 154.37 ppm, pH = 12.01, temperature 303 K) reached to equilibrium point in 80 min, which acquired for chemical adsorption-degradation reactions. The degredated SA dye data’s recorded by UV-visible spectroscopy for the occurrence of TMO-NMs of CuO-NPs at anticipated period of interval. The feasible performance of CuO-NPs was admirable, shows good adsorption capacity qm = 53.676 mg g^−1^ and most convenient to best fitted results establish by linear regression equation, corresponded for selected kinetic model (pseudo second order (R^2^ = 0.9981), equilibrium isotherm models (Freundlich, Langmuir, Dubnin-Radushkevich (D-R), Temkin, H-J and Halsey), and thermodynamic parameters (∆H° = 75461.909 J mol^−1^, ∆S° = 253.761 J mol^−1^, ∆G° = −1427.93 J mol^−1^, Ea = 185.142 J mol^−1^) with error analysis. The statistical study revealed that CuO-NPs was an effective adsorbent certified photocatalytic efficiency (η = 84.88%) for degradation of SA dye, exhibited more feasibility and good affinity toward adsorbate, the sorption capacity increases with increased temperature at equilibrium point.

Nanomaterials (NMs) are valuable constructive material in multidisciplinary science and have various applications due to their exclusive properties such as small size, larger surface area with volume, higher distortion of surface lattice energy and high thermal reactivity etc, which illustrate beneficial requirement for numerous purposes in a manner such as adsorption, catalysis, energy conversion storage, optoelectronics and drug delivery^1^,^2^,^3^,^4^,^5^. The organic dyes are the bigger macromolecules with different color, widely used in various industries for instance in textile, food, leather, cosmetic, plastic, leather etc. The average production of synthetic dyes from the industries is ~7 million tons worldwide. The removal of dyes in a smaller fragment molecule is a big issue for the environment protection, previously to solve this problem, well known different variety of waste agricultural material, metals, organic material are potentially applied through flocculation, coagulation and adsorption methods. It is realized that the applied methods were cost effective and not appropriate to destruct the bigger molecules of dyes, so the outcome was not satisfactory. To resolve this problem, recently the NMs, are one of the most and effective material, which participated to remove hazardous/toxicity of dyes in water^6^,^7^,^8^,^9^,^10^. In other words, NMs can solve the complications of environmental issues by reduction or degradation process in a cost effective manner^6^,^7^,^8^,^9^,^10^. Among various types of metal oxides (MOs) and transition metal oxide nanomaterials (TMO-NMs), CuO nanostructures, which is a p-type semiconductor, exhibit high photosensitivity with 1.21–1.5 eV energy band gap, showed better quantum efficiency, non-toxicity and performed as a photocatalyst for the deactivation of organic dye molecules mainly decomposes or deactivate via photocatalytic process and are more impulsive under UV-visible light^11^. The CuO nanostructures, emanates in transition metal series (TMS) and have unique and beneficial properties with ensurity in various field such as industrial, electronic, optoelectronic, sensor, biomedical and catalytic etc. Over various applications of CuO nanomaterials (CuO-NMs), it’s extensively utilized to clean the environment from organic, carcinogenic compounds, dyes, hazardous environmental contaminants^12^,^13^,^14^, through controlled photocatalytic process^15^. The CuO-NMs can be prepared via various physical and chemical techniques such as chemical vapour desposition (CVD), plasma enhance chemical vapour deposition (PE-CVD), surface mechanical attrition treatment (SMAT), metal organic chemical vapour deposition (MO-CVD), hydrothermal, sol-gel, spray pyrolysis, chemical solution process, solution combustion method^15^,^16^,^17^,^18^,^19^,^20^,^21^,^22^ etc.

The dyes are more hazardous and highly affected on human body system continuously. A series of diseases obtained from hazardous dyes reported in the literature such as hypertension, precordial pain, mental confusion, nausea, profuse sweating and methemo-globinemia, skin staining, dizziness, headache, Alzheimer, anemia, included eye burns problems are more terrible wound to see in organisms^23^,^24^,^25^,^26^,^27^. For healthy populations, establish clean environment, and it’s very important to understand the study of toxic substances (dyes) and their relations between biological species^28^,^29^. The organic dyes, which are toxic materials and affects to the flora and fauna at various stages. The CuO-NMs is a good photocatalyst and have strong reactivity to degradate various types of dyes such as methylene series red, orange and blue, acid orange 74 (an azo dye) by TPPO capped)^30^,^31^. Including the bare NPs of CuO, the doped materials with CuO was widely utilized to degradate dyes such as the organic compound acid black 1, was degradated via copper oxide-doped ZnO-NPs^32^,^33^. In another report, the chitosan was mixed with CuO-NPs and used to evaluate the photocatalytic property against MB dye. The copper oxide nano leaves (CuO-NLs) were implemented against the dye rose bengal and eosin Y dyes and was degraded successfully via photochemically^34^. There are so many conditions to define relative aptitude such as quantity or concentration of dyes, toxic effects duration & way to access, evaluate and control dyes, advanced materials and technique to control toxic effects, therefore, the fitness consideration of these points, analytical technique is fully supportive to detect, identify, quantify of toxic dyes, organic substances etc^35^,^36^. Due to fact its methodlogy very fast, simple, cheap, suitable to provides satisfactory result data’s for color and colorless routine laboratory samples analyzed via UV-visible spectrophotometer^37^. The small amount of NMs has ability to remove pollution due to their high strength with highly active sites^38^. In this direction, Antilen *et al*.^39^ explained the adsorption capacity of different type of NMs for the removal of heavy metals and dyes. For the measurement of adsorption capacity, efficiency, isotherm, kinetic parameter are employed and discussed in detail. On the basis of numerous potential applicability, surface thermodynamical properties are more effective to express thermodynamic consequences of NMs including thermodynamic parameters (ΔH°, ΔG°, ΔS°, and ΔE°) and kinetic models. The surface thermodynamic and kinetic models study showed great interest and significant to better understand the relationship between NMs and other foreign chemical species (reaction mechanism) by good adherence or adsorption properties. The adsorption kinetic study performed in an aqueous solution, which provides the valuable information for fixed number of sites of adsorbent and adsorbate material, with constant temperature. Numerous methods have been adopted to treat dye and waste water with various technologies such as biological, chemical and physical etc; these opted methods are very useful for the environmental purification. Literature survey reveals that the adsorption studies on various chemical substances were synthesized with the preferred material such as organic, inorganic, hybrid material, composite, which followed isotherm, kinetics, thermodynamics study for necessary assess adsorption and desorption manner. Basically, heterogeneous system favors adsorption techniques, which requires more than one ingredient, provides suitability, efficiency and easy to adopt the study of equilibrium and kinetics^40^. Including, these different error analysis (the residual root mean square error (RMSE), the chi-square test, sum of the square of the error (SSE), sum of absolute error (SAE), average relative error (ARE), average percentage errors (APE) and marquardt’s percent standard deviation (MPSD) were also evaluated and included here^41^.

The work reported here describes the formation of copper oxide nanoparticles referred to as CuO-NPs without using any catalyst with precursor copper acetate hydrate(Cu(CH_3_COO)_2_.H_2_O), n-propyl amine and NaOH through simple chemical precipitation process, temperature at ~90 °C in short time span (2 h). The general structural evaluation was investigated with field emission scanning electron microscopy (FESEM) and transmission electron microscopy (TEM) equipped with High resolution TEM (HR-TEM) arrangement whereas the crystalline property of the material was examined with X-ray diffraction pattern (XRD). In this manuscript, CuO-NPs were employed for the adsorption/degradation of safranine (SA) dye in an aqueous medium at pH 12.01 at regular time interval. The photocatalytic NPs was used as adsorbent to assess the adsorption capacity for sorption of SA dye. Here, the theme of our work is to investigate the parameters that influence initial solution pH, adsorbent dose, initial SA dye concentration and temperature. Our study provides the information related to collective results data’s such as adsorption kinetics, adsorption-equilibrium, adsorption thermodynamic with models and parameters at constant temperature, sorption study analyses via pseudo first and second order, Elovich model and intraparticle diffusion mechanism (IPDM). Inspite of these the equilibrium study was best fitted in Langmuir and Freundlich rather than Hasley, H-J, Temkin, and D-R model. The thermodynamic study to illustrate heat of adsorption at constant temperature, gives the value of thermodynamic parameters (ΔH°, ΔG°, ΔS°, ΔE°). Including these the performance of error analysis was also analyzed, which is based on mathematics describes the precision and accuracy. The percentage of smaller error is more accurate with good precise, calculate from calibration line. The data signifies that how dye molecule interact with the adsorbents. The best linear fit results provide the quality, which indicates the adsorption is monolayer or multilayer on homogenous or heterogeneous surface. The chemical interaction of NPs (CuO-NPs-SA) and dye adhesive surface properties were determine the sensitivity while kinetic, equilibrium, and thermodynamic properties exposed the behavior/activities with potential impact of designed CuO-NMs.

## Result and Discussion

### Morphological Characterization (FESEM& TEM) results

**Figure 1:**
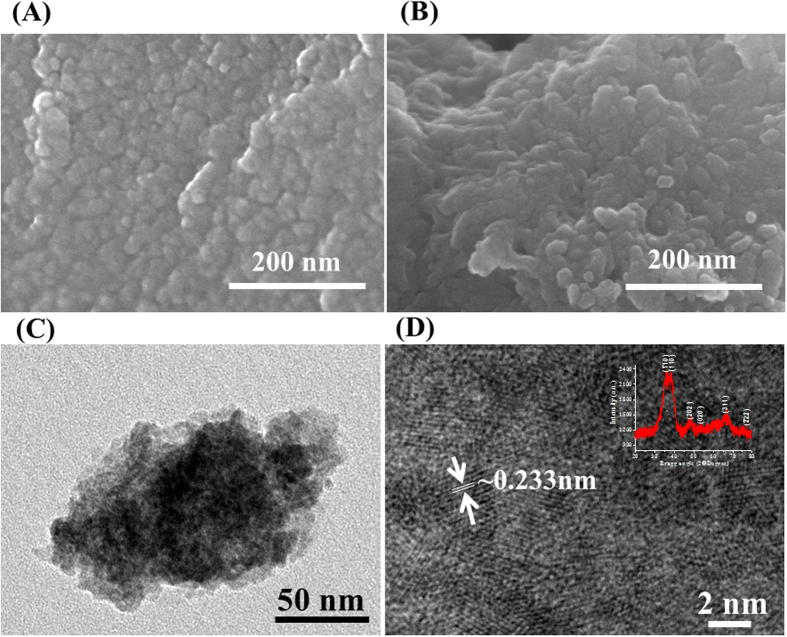
FESEM images of CuO-NPs. (**A**) Pure grown NPs, (**B**)After adsorption of NPs, (**C**)TEM image of clustered form of fine NPs, (**D**) The HR-TEM shows the lattice spacing between two fringes, which is ~0.233 nm. (Inset D) X-ray diffraction pattern of grown CuO-NPs.

To know the general morphology of the grown nanopowder sample of CuO-NPs, FE-SEM was utilized and the obtained results are displayed in Fig. 1. As can be seen from the obtained image, Fig. 1(A) shows that the as-prepared powder products having several aggregated molecules of NPs in scattered form. When the powder was checked in detailed (Fig. 1(A), confirmed that the prepared powder is in the range of nanoscale and possess in a very large quantity. The obtained powder is in spherical shaped tiny NPs, having an average size ~10 nm in range. Some particles are in aggregated form with the accumulation and conjugation of several spheres structures. From the image (Fig. 1(A)), the surface of obtained particles exhibit smooth, clean and spherical shaped structure. Another image Fig. 1(B) shows the particles after the adsorption of dye (SA) molecule, which clearly shows that the NPs are jointed with each other and arrange in a manner of an aggregated form of nanostructures due to adsorption process. Here, the used dye was completely entered in the pores and adsorb on the surfaces.

Further the morphological clarification was again confirmed via TEM at room temperature at an above parameter. Figure 1C shows the TEM image of NPs, clearly evident that the small NPs are gathered together and having small size of NPs. From TEM image (Fig. 1C), it is evident that the size of each NP diameter is in the range of ~10 nm, spherical, smooth surface, clearly consisted and justified with FE-SEM (Fig. 1A) observation. The HR-TEM image shows crystalline property of each NP, which shows that the NP is in the range of ~10 nm size spherical with aggregated manner. Due to very small in size, the clusters forms of NPs are merged together and show a section of aggregated molecule (Fig. 1C). The lattice space between two fringes was found ~0.233 nm (Fig. 1D), which corresponds to the commercial CuO-NPs^42^. The size, shapes and crystalline property (high resolution-TEM) of grown NPs observed from TEM analysis (Fig. 1C) are in consistent with the SEM images observation (Fig. 1A).

### X-ray diffraction spectroscopy (XRD)

The crystalline property of the grown nanomaterial evaluated through XRD as per the detailed in material in methods section. The XRD pattern shows peaks, which are similar to single crystalline CuO without impurities and matched with the standard JCPDS data card no. 05-661. The observed diffraction reflections in the pattern appearing at 35.45°, 3810°, 48.05°, 53.35°, 66.75°, 75.20° correspond to the lattice planes of (Ī11), (111), (202), (020), (311) and (222) respectively. The XRD spectrum shows the pattern related to the formation of NPs of CuO ((Fig. 1D inset)^3^.

The specific surface properties of the prepared NPs were examined in terms of area, volume and pore size distribution respectively. For this the BET surface area was analyzed, which was ~38.4 m^2^/g, Langmuir Surface Area was measured ~65.8 m^2^/g, whereas adsorption and desorption pore diameter were 45.3378 m^2^/g and 50.1026 m^2^/g respectively. The pore volume was analyzed at adsorption (0.124380 cm^3^/g) and desorption 0.124011 cm^3^/g conditions. Including these, the average pore diameter at adsorption and desorption conditions were about 109.7 A and 99.0 A respectively. It may assume that the opted chemically synthesized methodology for the preparation of NPs influence on the size of prepared NPs. The analyzed data supports that the NPs exhibit high porosity, which is the characteristic for various catalytic applications^3^,^4^,^42^.

### Photocatalytic evaluation of CuO-NPs against SA dye

**Figure 2:**
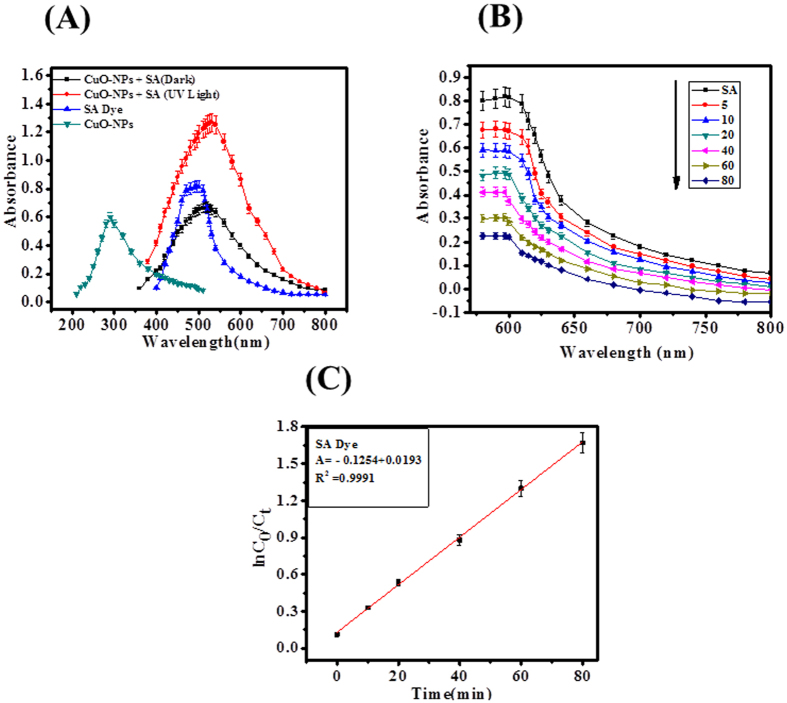
(**A**) Absorption spectra of SA dye with CuO-NPs, (**B**) Photocatalytic degradation of SA dye by CuO-NPs and (**C**) Linear plot of ln (C_0_/C_t_) vs time for photodegradation of SA dye.

To know the photocatalytic activity/degradation observation of SA dye with the prepared CuO-NPs, we have initially analyzed with UV-visible spectroscopy of blank SA dye and then CuO-NPs with SA dye (Fig. 2A). Obtained analysis shows that the grown NPs display outstanding degradation property against SA dye at 597 nm wavelength (Fig. 2(B)). For further, clarification reaction kinetics has been applied, which follows first-order kinetic and reaction rate kinetics was obtained as previously described (Fig. 2(C))^3^,^4^. The rate constant of CuO-NPs and safranine dye is 8.1 × 10^−2^ min^−1^ and efficiency of the catalyst was determined as equation 1 and it comes to 84.88%.


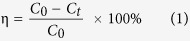


The photocatalytic degradation of safranine dye in aqueous suspensions is initiated by the photo- excitation of CuO-NPs, followed by the formation of electron-hole pair on the surface of the catalyst as equation 2. The direct oxidation of used dye reacts with the intermediates as equation 3, due to high oxidative potential of the hole (*h*_*VB*_^+^) in the catalyst.


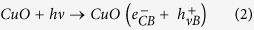



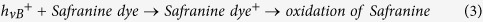


The photocatalytic activity or degradation of safranine dye was happens due to hydroxyl radicals (OH^−^) developed on the surface of NPs of CuO. The water molecule developed on the surface of NPs reacts with holes (*h*_*VB*_^+^) and forms the radicals of hydrogen and hydroxyl ions as equation 4. As the hydroxyl radicals (OH^−^) reacts with the whole of valance bond, which generates the hydroxyl ions as equation 5 & 6. It is postulated that the highest oxidation potential of OH^-^ radicals *(E0* =  +* 3.06 V)*, which is a main cause of mineralization of numerous organic dye molecules^37^,^39^.


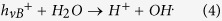



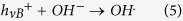



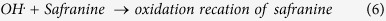


In photocatalytic degradation of safranine dye, the kinetic first order appreciates to degradation process under the influence concentration of safranine dye. The degradation rate constant value 8.1 × 10^−2^ min^−1^ obtained at initial concentration of safranine dye (1 × 10^−5^ M), indicates, increases the path length of photons which required in photocatalytic degradation process. The appropriate data from the linear graph (Fig. 2(C) corresponds to the rate law for the degradation reaction. The degradation rate constant is directly proportional to the probability of formation of hydroxyl radicals and reacted with SA dye molecules. The highest concentration of safranine molecules was adsorbed fixed amount of photons to support mobility of electron-hole and degradation efficiency (84.88%)^37^,^39^.

It is well known that the photocatalysis is a phenomenon of surface reactions, which depends on the existing surfaces for the reaction. The results of photocatalysis for CuO-NPs, explained on the basis of band gap (large) of prepared NMs, the hole and electron recombination delays the process, which results enhance the photocatalytic activity of the copper oxide nanomaterials^37^,^39^.

### Effect of pH on SA dye adsorption

**Figure 3:**
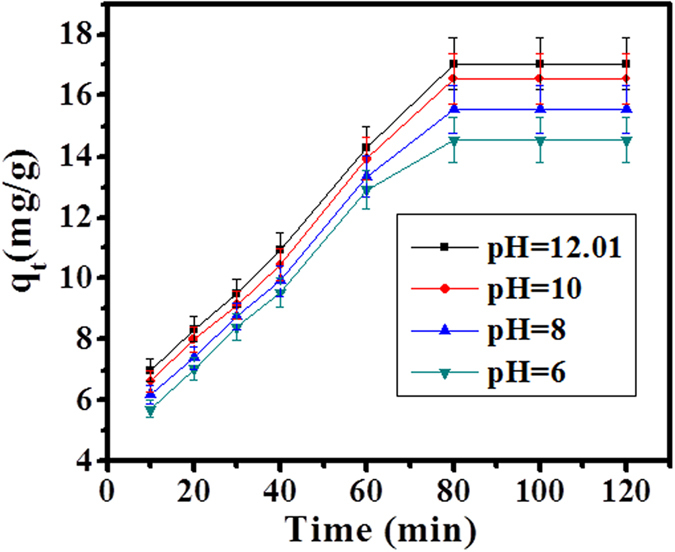
Effect of pH on SA dye adsorption by CuO-NPs at 303 K.

The maximum adsorption was analyzed on the surface of adsorbent in an aqueous solution, analytes (pH = 12.01 buffer solution (NaOH + HCl), 0.10 g CuO-NPs, 154.37 ppm conc. of SA dye and contact time 80 min at 303 K) were selected after optimization for adsorption process. The effect of pH, shown on adsorbent surface enhanced the adsorption process. The pH solution was affected on binding sites of adsorbent surface; this adsorption process enhanced chemical reaction between the molecules of SA dye on adsorbent particles via electrostatic interaction. As well as, increase logarithmic value of H^+^ or OH^−^ ions concentration with increase adsorption, because of the ionic strength of solution (positive and negative charge of adsorbent/adsorbate) was increases. As can be seen in Fig. 3. Shows that when the pH value increases from 6–12, it enhances the adsorption capacity with increase removal efficiency.

### Effect of CuO-NMs (adsorbent dose)

**Figure 4:**
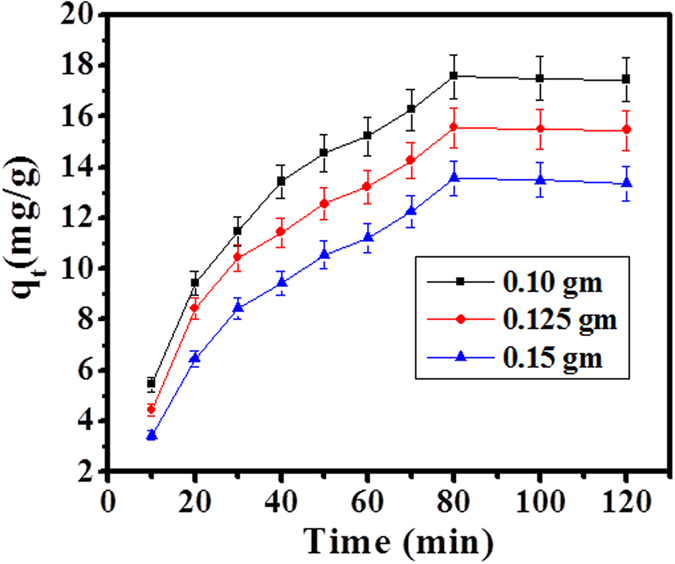
Effect of adsorbent dose of SA dye adsorption by CuO-NPs at 303 K.

In this experiment, the adsorption process is totally depending on particle size of adsorbent material exhibit larger surface area at quantified level. Due to small particle size of adsorbent molecule, it shows greater adsorption efficiency and sustains phenomena in an adequate performance. It is clear from the Fig. 4. Shows that as increases the amount of adsorbent material with increase adsorption efficiency but the per unit mass of adsorbate material decreases with decrease of adsorption density. Due to availability of number of active sites, which have larger surface area was responsible for greater adsorption.

### Effect of initial concentration of SA dye on CuO-NPs (adsorbent)

**Figure 5:**
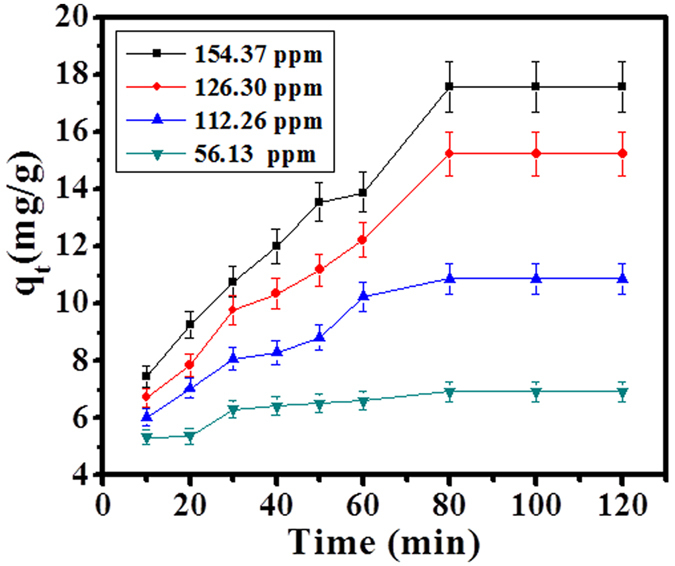
Effect of concentration of SA dye adsorption on CuO-NPs at 303 K.

As per the above explained adsorption procedure (0.10 g adsorbent, pH 12.01, 154.37 ppm dye solution with contact time 80 min at 303 K), intimated with four different concentration of SA dye solution (56.13, 112.26, 126.30 & 154.37 ppm) and the obtained result was shown in Fig. 5. The SA dye adsorption results indicate that the adsorption capacity gradually increases with increase concentration of SA dye. Finally the equilibrium reaches at maximum and constant concentration level at 154.37 ppm within time 80 min on adsorbent surface.

### Effect of temperature on adsorption process

**Figure 6:**
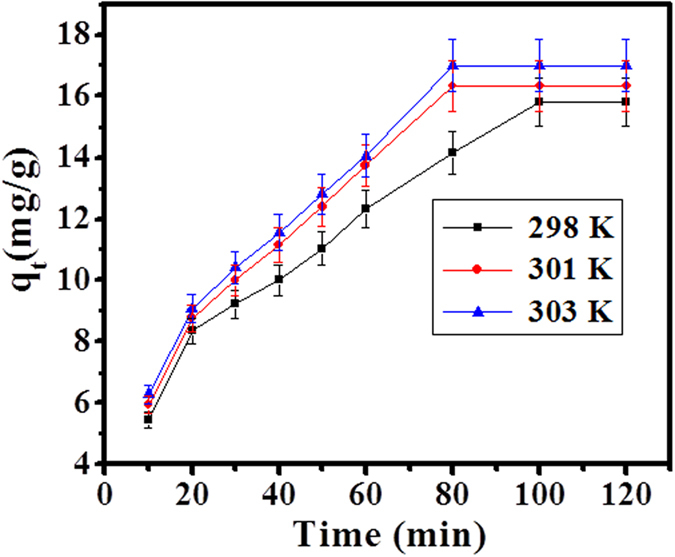
Effect of temperature on adsorption of SA dye by CuO-NPs at pH 12.01.

The adsorbent particle intake dye solution at varying temperature from 298, 301, and 303 K on equilibrium. It is apparent from Fig. 6. that as the temperature increases; it also enhances the adsorption process, which exhibited that the adsorption reaction is endothermic and spontaneous. As the adsorption efficiency increases with increases of dye molecules mobility or intraparticle diffusion rate of SA dye, it more and more interacted towards active sites of adsorbent molecule via increases in temperature, but it decrease viscosity and activation energy.

### Kinetic model

The adsorption process rate expression defined by kinetics reaction in mathematical terms, frequently adsorption rates is dependent on the surface area of nano-adsorbent. In adsorption process applied for four kinetic models such as pseudo first order equation, pseudo second order equation, Elovich equation and intraparticle diffusion equation, which was moderately different to each other. The kinetic study, Lagergren pseudo first order, Ho and McKay pseudo second order, Elovich and intraparticle diffusion models were used to test the experimental data^43^,^44^,^45^,^46^.

#### Lagergren pseudo first order kinetics

The pseudo-first-order equation is generally expressed as follows^43^:


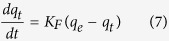


Where *q*_*t*_ is the amount of adsorbate adsorbed (mg g^−1^) at time t, q_e_ the adsorption capacity at equilibrium (mg g^−1^), *K*_*F*_ the pseudo-first-order rate constant (min^−1^), and ‘t’ the contact time (min). The integration of Eq. 7 with the initial condition, *q*_*t*_ = 0 at *t* = 0 and *q*_*t*_ = *q*_*t*_ at *t* = *t*, the equation becomes:


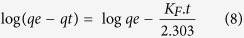


A plot of log (*q*_*e*_ − *q*_*t*_) versus time, ‘t’ gives the value of *K*_*F*_ and *q*_*e*_ from slope and intercept respectively.

#### Ho and McKay Pseudo second order kinetic model

The pseudo second order model is represented as^44^:


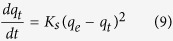


Where *K*_*s*_ is the pseudo second order rate constant (g mg^−1^ min^−1^). Integrating Eq. 9 and applying boundary conditions, *q*_*t*_ = 0 at *t* = 0 and *q*_*t*_ = *q*_*t*_ at *t = t*, the equation becomes:


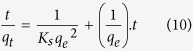


A plot between *t/q*_*t*_ versus *t* give the values of pseudo second order constant *K*_*s*_ (g mg^−1^ min^−1^) and *q*_*e*_ (mgg^−1^). The initial sorption rate, *h* (mg g^−1^ min^−1^), at t → 0 is defined as:


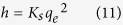


From Eqs (10) and (11) we get the following new equation


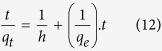


The initial sorption rate *h* is obtained from the intercept since *q*_*e*_ known from the slope, the second order rate constant K_s_ can be determined from the value of *h.*

#### The Elovich equation

The Elovich equation is symbolized as^45^:


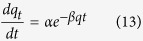


Integrating this equation for the boundary conditions, gives:


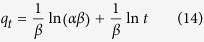


Where *α* (mg g^−1^ min^−1^) is the initial sorption rate and *β* (g mg^−1^) is related to the extent of surface coverage and the activation energy involved in chemisorption.

#### Intraparticle diffusion equation

The intra particle diffusion equation can be explored as^46^:


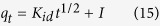


where *K*_*id*_ (mg g^−1^ min^−1/2^) is the intra particle diffusion rate constant.

**Figure 7:**
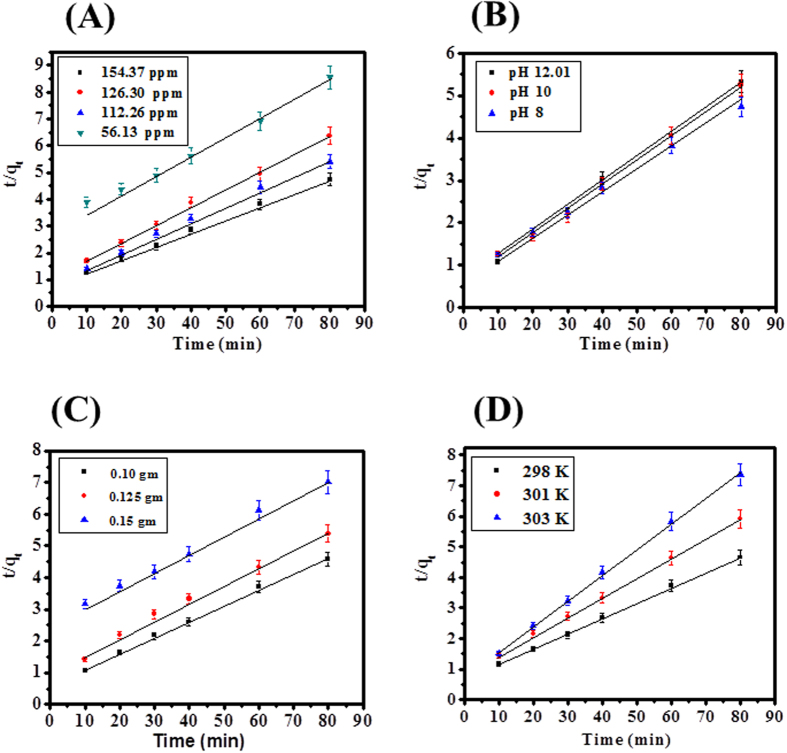
(**A**) Pseudo second order kinetic model for different concentrations of SA dye, (**B**) Pseudo second order kinetic model for adsorption of SA dye at different pH, (**C**) Pseudo second order kinetic model for adsorption of SA at different adsorbent dose and (**D**) Pseudo second order kinetic model for adsorption of SA dye at various temperatures.

A plot of *q*_*t*_ versus *t*^1/2^ is presented for the adsorption of SA dye onto CuO-NPs. Values of *I* gives an idea about the thickness of the boundary layer, i.e., the larger the intercept, the greater is the boundary layer effect. As explained above four different models (pseudo first, pseudo second, Elovich and Intraparticle diffusion) were employed and set the experimental data’s and obtained results are explained in the form of linear regression equation. The graph plot between *t/q*_*t*_ versus *t* as can be shown in Fig. 7(A–D).

Kinetics parameters are supported to pseudo second order at 154.37 ppm concentration of SA dye, pH = 12.01, 0.10 g of adsorbent at temperature 303 K. The validity order of adsorption was checked and calculated by kinetic parameters such as regression coefficients (R^2^), *qe* (mg/g), value of K_2_ (g mg^−1^ min^−1^), h (mg g^−1^ min^−1^). The values of all parameters are showed in Table S1. In our experiment, we have found that the values of *R*^2^ (0.9981) was highest for pseudo second order and greater then all adsorption kinetic models such as pseudo first order (0.9979), Elovich model (0.9826) and Intraparticle diffusion model (0.9523).

In our experiments SA dye adsorption order follows as: (highest Pseudo second order and lowest Intraparticle diffusion model).


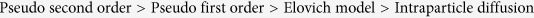


The values of k_2_ = 0.00404 (g mg^−1^ min^−1^), qe_cal_ = 19.9481 (mg g^−1^), h = 1.6088 = (mg g^−1^ min^−1^) for pseudo-second-order, K_1_ = 0.0034 (min^−1^), qe_cal_ = 16.9824 mg g^−1^ for pseudo-first-order, α = 2.9472 mg/g min), β = 0.2646 g/mg) for Elovich model, k = 1.3314 (mg g^−1^ min^− 1/2^), I = 6.060 (mg/g) for intraparticle diffusion modal calculated at 303 Kelvin temperature. Pseudo-second-order increases temperature (298–303 K), increases adsorption capacity (q_e_) with increases adsorption rate constant K_2_ (g mg^−1^ min^−1^) (Table S1). All data values are denoted to fixed concentration of SA dye (154.37 ppm), increases temperature (298–303 K), increases adsorption capacity (q_e_) with respect to adsorption rate constant K_2_ (g mg^−1^ min^−1^) (Table S2).

### Isotherm model

Generally, adsorption isotherm characterize to equilibrium data of adsorption process, which designed by optimized procedure and mention to relationship between surface of adsorbent material and amount of adsorbate dye molecules, transferred on active sites in an aqueous phase. The adsorption equilibrium data were analyzed by the Freundlich, Langmuir, Dubinin–Radushkevich, Temkin, Harkins-jura and Hasley isotherm models and their parameters resolved aspect of adsorption process. All these equation parameters are frequently advised for adsorption mechanism and surface properties of bonded electron materials affinity with thermodynamic assumptions^45^,^47^,^48^,^49^,^50^,^51^,^52^,^53^.

#### Langmuir adsorption isotherm expressed by linear form equation:


**Figure 8:**
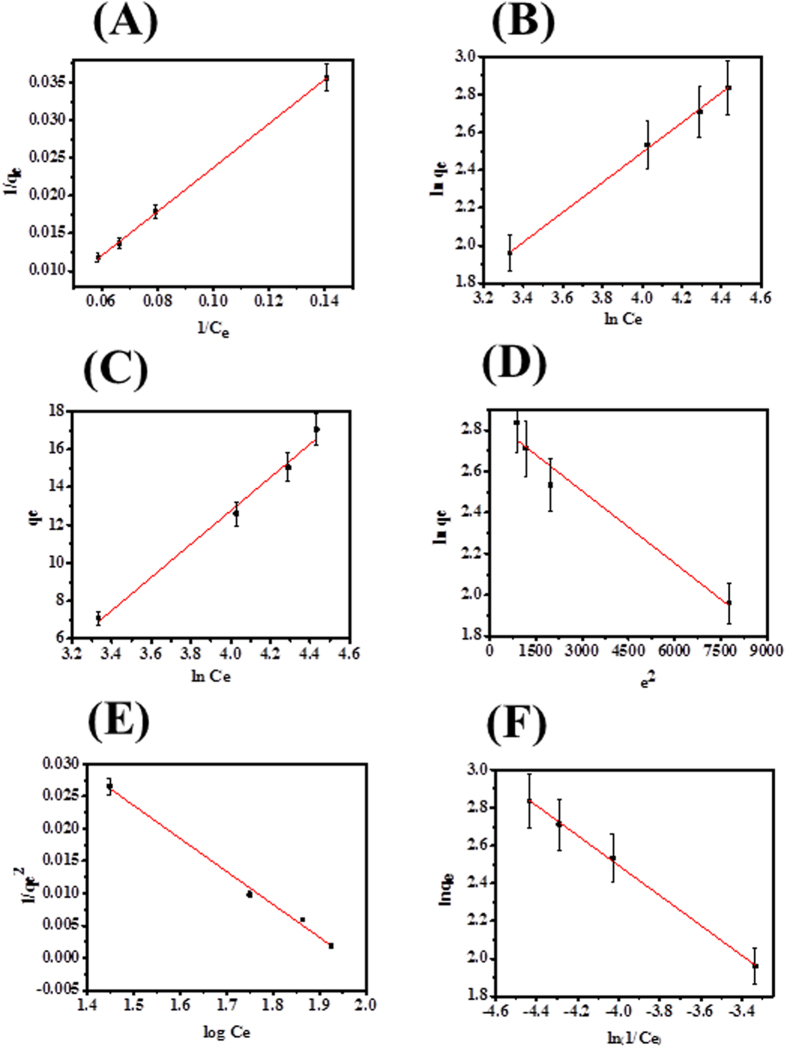
Different types of Adsorption isotherm (Langmuir (**A**), Freundlich (**B**), Temkin (**C**), D-R (**D**), H–J (**E**), Halsey (**F**) for SA dye in pH 12.01 at 303 K.

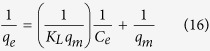


where qe (mg g^−1^) equilibrium concentration of CuO-NPs, Ce (ppm) equilibrium concentration of SA dyes, K_L_ is Langmuir constants; q_m_ is adsorption capacity of monolayer of adsorbent (mg/g) respectively. The values of q_m_ and K_L_ are calculated from the plot between 1/q_e_ versus 1/C_e_ as can be seen from Fig. 8(A). Determined from the intercept and slope (Table S3). The R_L_ is the dimensionless equilibrium parameter of the Langmuir model and expressed by the equation^47^:


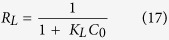


where K_L_ is the Langmuir constant (L mg^−1^) and C_0_ is the initial concentration of SA dye (ppm).

The adsorption isotherm is favorable (0 < R_L_ < 1), unfavorable (R_L_ > 1), linear (R_L_ = 1) and irreversible (R_L_ = 0), which depend on separation factor R_L_. In this study, the value of R_L_ (0.230–0.546) indicates favorable adsorption for SA dye concentration (154.37) ppm at 303 K.

#### Freundlich isotherm^48^

Freundlich isotherm model presumed for heterogeneous surface and exponentially distributed adsorption sites of adsorbate on adsorbent surface. The linear equation of Freundlich isotherm is presented below as exponential form:


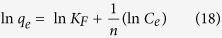


where *q*_*e*_ is the dye molecules adsorbed at equilibrium time, *C*_*e*_ is the equilibrium concentration of dye molecules in solution, *K*_*F*_ (L g^−1^) and ‘n’ are Freundlich constants characteristics of the system, indicating the adsorption capacity and adsorption intensity, respectively. The isotherm constant can be calculated from the intercept and slope of plot between *lnq*_*e*_ versus *lnC*_*e*_ Fig. 8(B). The values of Freundlich parameters (*K*_*F*_, n and R^2^) are presented in Table S3 indicates that the favorable adsorption of SA dye 154.37(ppm) at 303 (K) temperature adsorbate on adsorbent surface was obtained.

#### Temkin isotherm model

Temkin isotherm model understand that linear form is better for adsorption rather than the logarithmic, gradually reduces heat adsorption of molecules or ions linearly to adsorbent-adsorbent surface interaction^49^.


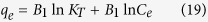


where *K*_*T*_ is the equilibrium binding constant (L g^−1^) related to the maximum binding energy, *B*_*1*_ is the heat of adsorption, the plot of *q*_*e*_ versus *lnC*_*e*_ facilitate the determination of isotherm constants *B*_*1*_ and *K*_*T*_ from the slope and intercept, respectively for adsorption of SA dye on CuO-NPs is presented in Fig. 8(C). If the value of *B*_*1*_ constant is less than 8 kJ/mol, its means a weak interaction between adsorption-adsorption surfaces was formed, therefore value of *B*_*1*_ constant found to be 287.018 KJ/mol, proved feebled interaction in between SA dye molecules and CuO-NPs Table S3.

#### D-R model isotherm

D-R model isotherm was approached for adsorption via the linearized equation form show as below^50^:


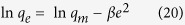


where β is a constant related to the mean free energy of adsorption per mol of the dye (mol^2^ kJ^−2^) when it is transferred to the surface of the adsorbent from infinity in solution, q_s_ is the theoretical saturation capacity, e is the Polanyi potential given as:


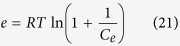


where R is the universal gas constant (8.314 J/mol K), T (K) is temperature.

By making certain assumptions, the mean energy of adsorption E calculated from the b value employing, the relation


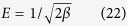


The graph is plotted between *lnq*_*e*_ versus *e*^*2*^, *β* and *q*_*m*_ both values are calculated from slope and intercept of linear plot Fig. 8(D). The mean of energy adsorption (E) value is 54.124 (J/mol) onto adsorbent surface of CuO-NPs at 303 K.

#### Harkins-jura isotherm

Harkins-jura isotherm model was establish for multilayer adsorbent surface which exhibit heterogeneous pore, distributed on non-uniform surface and adjacent site apart from each other. The following expression of Harkins-jura equation as^51^:


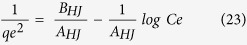


where B_HJ_ and A_HJ_ are the isotherm constants and both constant values are calculated from the slope and intercept of the plot. The graph was plotted between 1/qe^2^ and log Ce as seen in Fig. 8(E). The appropriate isotherm parameters values are summarized in Table S3.

#### Halsey isotherm

Halsey isotherm model appropriate to multilayer adsorption at a quite big distance from the surface of heterogeneous adsorbent and equation expression is given as^52^:


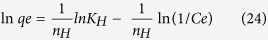


where n_H_ and K_H_ are Halsey isotherm constants, the values of both constant are given in Table S3 and obtained from the slope and intercept of the linear plot of ln q_e_ against ln (1/C_e_) Fig. 8(F).

The precise all parameters of adsorption isotherms (Langmuir, Freundlich, Hasley, Temkin, D-R Model, H-J model) are reported in Table S3. The linear square regression correlation coefficient value was obtained from the slop and intercept of each isotherm plot. As compared to correlation coefficient values, the higher value for Langmuir isotherm adsorption model (R^2^ = 0.9996) and minimum (R^2^ = 0.9490) for D-R isotherm adsorption model was observed. Therefore, the higher value of R^2^ is clearly shows that Langmuir isotherm adsorption model was best fitted in sorption with maximum adsorption capacity q_m_ = 53.676 (mg g^−1^). The adsorption isotherm models are followed in a manner:


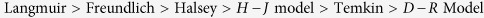


The evidence of experimental data’s was adequate with respect to adsorption of SA dye onto CuO-NPs and such adsorption mainly occurred on the heterogeneous surface of the adsorbent.

### Error analysis

To determine the error data analysis of equilibrium isotherm models by the linear approach, which gives minor error data values to indicate best fit in isotherm models. The calculated values of isotherm errors such as Residual root mean square error (RMSE), chi-square test, sum of the squares of the errors (SSE), average relative error (ARE), and sum of the absolute errors (SAE), average percentage errors (APE), Marquardt’s percent standard deviation (MPSD) are summarized in Table S4. After calculation each isotherm parameters provided minimum error values with respect to order of significance concentration error functions.

### Activation energy

Activation Energy is the minimum amount of energy that require to activate the molecules in sorption process. The activation energy is the difference of final and initial between atoms or molecules in an activeted/transition-state. The activation energy *E*_*a*_ is determined as per the Arrhenius equation^45^:


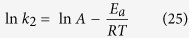


where k_2_ is the rate constant value for the SA dye, *E*_*a*_ is the activation energy in kJ mol^−1^, T is absolute temperature, R is gas constant and A is constant called frequency factor. Value of *E*_*a*_ can be determined from the slope of lnk_2_ versus 1/T plot (Fig. S1). From the plot the value of *E*_*a*_ was found to be 185.142 kJ mol^−1^. The obtained positive of *E*_*a*_ advocates that, when the temperature increases it enhances the adsorption process and illustrates the nature of the adsorption process is endothermic.

### Thermodynamic evaluation of the process

The change of adsorption process at equilibrium level is the Gibbs free energy, which defined by the classic Van’t Hoff as^45^:


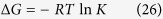


The Gibbs free energy is also related to the entropy change and heat of adsorption at constant temperature by following equation:


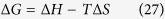



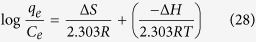


where qe the metal ion adsorbed (mg g^−1^), Ce is the equilibrium concentration (ppm), T is temperature (Kelvin) and *q*_*e*_*/C*_*e*_ is called adsorption affinity. The above equation is for unit mass of adsorbent dose.

The Gibbs free energy (∆G°) of adsorption process relies with their parameters such as enthalpy change (∆H°), entropy change (∆S°). The condition of ion-exchange process (adsorption process) is spontaneous or not deliberated by thermodynamic conditions. Therefore, the value of ∆H° and ∆S° calculated from slope and intercept value of graph plot of log *(q*_*e*_*/C*_*e*_) versus 1/T for Gibbs free energy shown in Fig. S2. The values of ∆G°, ∆H°, ∆S° are calculated at 298, 301 and 303 K (Table S5). The negative value of ∆G° indicates that the adsorption process is spontaneous, positive value of ∆H° and ∆S° shows adsorption process is endothermic at increased temperature (Table S5).

## Conclusion

In this manuscript, fix or quantified substances were used in sorption process and selected by optimized reaction parameters such as pH = 12.01, concentration 154.37 ppm SA dye, 0.10 g adsorbent amount and temperature 303 K. The maximum adsorption capacity of SA dye was adsorbed on CuO-NPs found to be highest at 303 K in pH=12.01 medium with equilibrium time at 80 min. The kinetic model shows that the pseudo-second-order model is more connivance with R_L_ values (0.230–0.546) suitable to favorable adsorption of SA dye. Beside this, the adsorption equilibrium model was illustrated by Langmuir, Freundlich, D-R, Temkin, H-J and Hansley isotherm models but in all these models, one model which is best fitted in adsorption of SA dye onto the CuO-NPs is Langmuir model (R^2^ = 0.9996), obtained the values of adsorption capacity of SA dye 53.676 mg g^−1^. The statistical error data’s analysis provides suitable and accurate description of the experimental equilibrium data. Therefore, the order of isotherm parameters to fit and used linearization from linear regressive equation for selects the best error function. The negative values of ∆G° showed that feasible and spontaneous, positive ∆H° and ∆S° values depicted endothermic nature with increased arbitrariness at the solid-solution interface of the sorption of SA dye onto CuO-NPs. The activation energy of adsorption evaluated with the second order rate constants.

## Material and Methods

### Experimental details

#### Synthesis of catalyst free copper oxide CuO-NMs

The formation of CuO-NPs was accomplished with the use of precursor copper acetate hydrate (Cu(CH_3_COO)_2_.H_2_O), n-propyl amine and sodium hydroxide (NaOH) purchased from Aldrich chemical corporation USA and used as received. In an experiment: 15 mM copper acetate hydrate (~300 mg) and n-propylamine (20 mL) were mixed in 100 mL of methanol (MeOH) under constant stirring, after mixing blue colored solution was appeared in a beaker. To this transparent blue colored solution, NaOH (2 × 10^−1^ M, ~200 mg), mixed and shaked each time for complete mingling. After the complete addition, pH of solution was checked via pH meter (cole parmer, USA). Due to increase basicity of the solution, pH was reached upto 12.01. After the complete mixing, the solution was shifted to glass pot with two necked arrangement and heated at 90 °C for 2 h. As the solution temperature rises, the blue colored solution changes to dark brown colored solution and then black. After refluxing, the precipitate of the formed powder product was steady to the glass pot. Washed well with alcohol and characterized as previously described^6^.

#### Characterizations of CuO-NMs

The X-ray diffraction (XRD, Rigaku, Japan) pattern was utilized to know the particle size, phase and crystalline property of the precipitated material in the range from 20–80° in Cu_Kα_ radiation (λ = 1.54178 Å) with 6°/min scanning speed with an accelerating voltage of 40 kV and current of 40 mA. The general morphology of the prepared NPs was examined via filed emission scanning electron microscopy (FESEM, Hitachi S-4700, Japan) at room temperature. To know the detailed observation of SEM, the dried powder was uniformly scattered on carbon tape and coated with thin conducting layer of platinum (pt) for 3 seconds (sec). Further the clarification related to morphology of the prepared NPs, powder was again analyzed with TEM (JEOL JEM JSM 2010 at 200 kV, Japan). Including these the particles average BET, Langmuir surface area, Adsorption, desorption pore size distribution for prepared sample (~0.10 g) was examined by using instrument ASAP 2010 (mentics, USA), under N_2_ atmosphere with degassing temperature 250 °C overnight.

#### Photodegradation of CuO-NPs

The degradation of safranine dye in presence of prepared CuO-NPs was carried out in a special type of glass pot/reactor (250 mL capacity) designed for photocatalytic evaluation as described previously^4^,^36^. The detailed estimation of degradation of dye, ~5 mg NPs of CuO was mixed with 1 × 10^−5^ M of safranine dye in 100 mL capacity distilled water (DW) with uninterrupted stirring condition. Initially, the controlled or blank experiment was performed to display that no reaction was takes place in absence of UV-light. Each time ~5 mL sample was extracted to remove the catalyst by ultra-centrifuge (3000 rpm/min) process earlier the observation of UV-vis data. The photocatalyzed or UV-light exposed sample was collected from the glass pot (250 mL capacity) at regular time intervals^4^,^36^.

### Sorption procedure

The sorption process was performed in a 100 mL of conical flask using SA dye with CuO-NPs under ambient conditions. The SA dye concentrations range from 56.13 to 154.37 ppm was opted. The amount of adsorbent (0.10 g) CuO-NPs was added to SA dye solution concentration under stirring at 90 rpm at 303 K at constant pH of 12.01 for 1 h to reach their equilibrium point. The experimental solution was sampled at regular time interval. After complete mixing of adsorbent and dye solution, samples were centrifuged for 10 min and collected the filtrate. The effect of parameters such as effect of pH, time, temperature, adsorbent dose and dye concentration were checked in solution phase. The supernatant solution concentrations of SA dye were analyzed by UV visible spectrophotometer (Shimazu, Japan, UV-2555) at maximum wave lengths of 597 nm. The adsorbed amount of SA dye at equilibrium (mg g^−1^) was calculated by the following expression^53^:


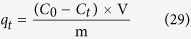


where q_t_ (mg·g^−1^) is the amount adsorbed per gram of adsorbent, C_0_ (ppm) and C_t_ (ppm) are the concentration in solution at time t = 0 and at time t, V is the volume of solution (L) and ‘m’ is the mass of adsorbent (g).

#### Effect of pH on SA dye sorption

The influence of pH on amount of used adsorbent CuO-NPs was analyzed at different pHs from 6, 8, 10 and 12 respectively. The adsorbent amount from 0.10 g was utilized for the whole study in solution form. In this experiment the prepared adsorbent solution was filled in a specialized conical flask and placed on a rotatory water bath and shaked at a fixed speed from temperature at 298, 301 and 303 K at 90 rpm for a period of 4 h. After the centrifugation, the samples were decanted at different time intervals in equilibrium time period.

#### Effect of concentration (SA Dye) on adsorption

The different concentrations (56.13, 112.26, 126.30 and 154.37 ppm) of SA dye were selected to investigate the effect of concentration of dye onto prepared CuO-NPs. The data were observed in terms of adsorption capacity (q_t_) verse time (t). As the concentration of dye increases in the solution with increase adsorption process.

#### Effect of adsorbent dose

For adsorption, the adequate amount of CuO-NPs selected maximum uptake of dye molecule in range from 0.10 to 0.15 g. The surface area of adsorbent material doses increase with increase of adsorption of SA molecules. Consequently, adsorption reaches at equilibrium phase selected at 0.10 g adsorbent for the whole experiment.

#### Effect of temperature on adsorption

In this experiment adsorption of dye (154.37 ppm) on CuO-NPs (0.10 g) at different temperature from 298, 301 and 303 K respectively, was checked. As the temperature increases, the adsorption of dye (SA) molecule increases on adsorbent surface.

#### Error analysis

The following error functions were to determine isotherm parameters (Freundlich, Langmuir, Temkin, D-R, H-J and Halsey) most suitable and appropriate results found to be linearization form of isotherm model, which represented the experimental data’s. The fascinating mathematical approach was described the adsorption isotherms at a constant temperature for dye adsorption and calculates the whole sorption behavior under different operating conditions^54^,^55^,^56^,^57^.

**Residual root mean square error (RMSE):**


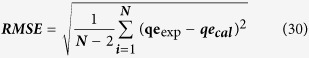


**The chi-square test:**


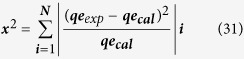


**The Sum of the squares of the errors (SSE):**


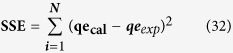


**The average relative error (ARE):**


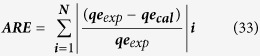


**The sum of the absolute errors (SAE):**


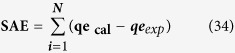


**The average percentage errors (APE):**


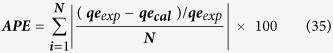


**Marquardt’s percent standard deviation (MPSD):**


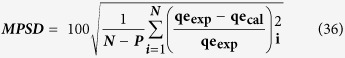


### Equilibrium isotherm

The equilibrium kinetics was evaluated at different concentration of adsorbent ranging from 56.13 to 154.37 ppm. The fix amount of adsorbent molecule was added in each of conical flask and the obtained suspension solution was mixed at 90 rpm with constant pH of 12.01 for 4 h. The amount of ions presents in the suspension was determined titrimetrically as explained above^47^,^48^,^49^,^50^,^51^,^52^.

### Kinetics model

To investigate the adsorption study with CuO-NPs, different kinetics models such as Lagergren pseudo first order, Ho and McKay pseudo second order, Elovich model and intraparticle diffusion models were used to test and evaluate, controlling adsorption process the experimental data^43^,^44^,^45^,^46^.

### Thermodynamic study

The synthesized CuO-NPs were used with SA dye (154.37) in a conical flask and shaked at 90 rpm on water bath, and separated via centrifugation. After the optimization (conc. of SA dye solution, pH buffer, adsorbent dose and temperature) were determined and calculated adsorbed amount of SA dye at equilibrium *qe* (mg.g^−1^)^45^.

## Additional Information

**How to cite this article:** Wahab, R. *et al*. Photocatalytic TMO-NMs adsorbent:Temperature-Time dependent Safranine degradation, sorption study validated under optimized effective equilibrium models parameter with standardized statistical analysis. *Sci. Rep.* **7**, 42509; doi: 10.1038/srep42509 (2017).

**Publisher's note:** Springer Nature remains neutral with regard to jurisdictional claims in published maps and institutional affiliations.

## Supplementary Material

Supporting Information

Supplementary Figure S1

Supplementary Figure S2
