# Supplementary material for: Photocatalytic TMO-NMs adsorbent: Temperature-Time dependent Safranine degradation, sorption study validated under optimized effective equilibrium models parameter with standardized statistical analysis
Source: Sci Rep. 2017 Feb 14;7:42509. doi: 10.1038/srep42509 (PMC5307350; doi:10.1038/srep42509)
Supplement: Supporting Information [file srep42509-s1.pdf]

Supporting Information

Photocatalytic TMO-NMs adsorbent: Temperature-Time dependent Safranine degradation, sorption study validated under optimized effective equilibrium models parameter with standardized statistical analysis

Rizwan Wahab[1,2†]*, Farheen Khan[3†], Nagendra Kumar Kaushik[4], Javed Musarrat[5] and Abdulaziz A.Al-Khedhairy[1]

[1]*Zoology department, College of Science, King Saud University, Riyadh 11451, Saudi Arabia*

[2]*Al-Jeraisy, Chair for DNA Research, Department of Zoology, College of Science, King Saud University, Riyadh 11451, Saudi Arabia*

[3]*Department of Chemistry, Aligarh Muslim University, Aligarh U.P. India*

[4]*Plasma Bioscience Research Center, Kwangwoon University, Seoul 139701, South Korea*

[5]*Dept.of Ag.Microbiology, AMU, Aligarh, India,* [6]*Baba Gulam Shah Badshah University, Rajouri, J&K, India.*

[†]These authors have contributed equally to this work.

***Corresponding Authors:** RW (rwahab@ksu.edu.sa)

Supporting Table

Table S1. Different type of kinetic models for the adsorption of safranine dye 154.37 ppm.

Temperature	qe $_{exp}$ (mg g^{-1})	Pseudo first order			Pseudo second order			
		k_1 (min^{-1})	qe $_{cal}$ (mg g^{-1})	R^2	k_2 (g mg^{-1} min^{-1})	qe $_{cal}$ (mg g^{-1})	h (mg g^{-1} min^{-1})	R^2
**298K**	11.7462	0.0079	9.4297	0.9915	0.00121	17.8635	0.3881	0.9965
**301K**	15.3638	0.0044	14.3879	0.9945	0.00280	18.3418	0.9442	0.9945
**303K**	17.4821	0.0034	16.9824	0.9979	0.00404	19.9481	1.6088	0.9981

Elovich	α (mg/g min)	β (g/mg)	R^2	Intraparticle Diffusion	k (mg g^{-1} $min^{-1/2}$)	I (mg/g)	R^2
**298K**	0.1672	0.2050	0.9476	298K	1.8163	1.6880	0.9675
**301K**	2.2347	0.2565	0.9752	301K	1.3966	2.9778	0.9853
**303K**	2.9472	0.2646	0.9826	303K	1.3314	6.0608	0.9523

Table S2. Pseudo-second-order kinetic model parameters for the adsorption of safranine dye.

S. No	System parameter	k_2 (g mg^{-1} min^{-1})	q_e (mg/g)	h (mg/g min)	R^2
**Initial SA dye concentration pH 12 at 303K**					
**1.**	56.13 ppm	0.00148	1 4.905	0.3301	0.9912
**2.**	112.26 ppm	0.00401	15.124	0.9334	0.9970
**3.**	126.30 ppm	0.00364	17.364	1.1011	0.9959
**4.**	154.37 ppm	0.00404	19.9481	1.6088	0.9981
**Initial solution pH at 303K**					
**5.**	6	0.00684	16.697	1.9094	0.9962
**6.**	10	0.00625	17.137	1.8374	0.9934
**7.**	12.01	0.00404	19.9481	1.6088	0.9981
**Amount of adsorbent in pH 12.01 at 303K**					
**8.**	0.1g	0.00404	19.9481	1.6088	0.9981
**9.**	0.125g	0.00288	18.158	0.9504	0.9920
**10**	0.15g	0.00123	17.733	0.3888	0.9942
**System Temperature in pH 12**					
**11.**	25°C	0.00936	11.958	1.3387	0.9990
**12.**	28°C	0.00336	15.827	1.1875	0.9995
**13.**	30°C	0.00404	19.9481	1.6088	0.9981

Table S3. Parameters of different types of isotherms for safranine dye at 154.37 ppm.

S. No	Isotherm	Temperature	Parameters			
			K_f	n		R^2
1.	Freundlich	298	1.8185	2.3163		0.9847
		301	1.3009	1.4187		0.9897
		303	0.8563	1.2609		0.9982
2.	Langmuir		K_L (L mg^{-1})	q_m (mg g^{-1})		R^2
		298	0.02182	13.475		0.9995
		301	0.00744	34.470		0.9993
		303	0.00542	53.676		0.9996
3.	Temkin		K_T	b		R^2
		298	0.0465	628.651		0.9326
		301	0.0275	367.390		0.9686
		303	0.0230	287.018		0.9870
4.	D-R		q_m	β	E (J/mol)	R^2
		298	9.4130	1.1706×10^{-4}	85.094	0.9054
		301	14.6828	1.2705×10^{-4}	62.731	0.9201
		303	17.3946	9.605×10^{-5}	54.124	0.9490
5.	H-J		A_{HJ}	B_{HJ}		R^2
		298	16.1603	0.1122		0.9250
		301	17.4520	0.1326		0.9167
		303	19.6232	1.9627		0.9917
			K_H	n_H		R^2
6.	Hasley	298	0.5769	2.3163		0.9408
		301	0.4650	1.4250		0.9897
		303	0.4250	1.2611		0.9982

Table S4. Error analysis.

S.No	Isotherm	Temperature (K)	RMSE	X^2	SSE	ARE	SAE	APE	MPSD
**1.**	**Langmuir**	303	0.0847	0.0008	0.0143	0.0070	0.1199	0.1759	5.932
**2.**		301	0.1971	0.00005	0.0777	0.0195	0.2788	0.4877	9.878
**3.**		298	0.0160	0.00005	0.0005	0.0023	0.0227	0.0584	208.64
**4.**	**Freundlich**	303	0.0813	0.0007	0.0132	0.0067	0.1143	0.1677	2.895
**5.**		301	0.3385	0.0155	0.2292	0.0335	0.4788	0.8377	2.369
**6.**		298	0.1936	0.0075	0.0749	0.0282	0.2738	0.705	1.994
**7.**	**Temkin**	303	0.2940	0.0104	0.1729	0.0244	0.4159	0.6103	7.125
**8.**		301	0.2566	0.0094	0.1317	0.0254	0.363	0.6352	6.791
**9.**		298	0.2822	0.0157	0.1593	0.0041	0.3992	0.0010	9.059
**10.**	**D-R**	303	1.594	0.2634	5.0823	0.1323	2.254	3.308	25.722
**11.**		301	1.1624	0.1696	2.7027	0.1150	1.644	2.877	119.937
**12.**		298	0.082	0.0013	0.0136	0.0120	0.117	0.301	7.7624
**13.**	**H-J**	303	11.731	618.243	275.241	0.9738	16.590	24.346	69.780
**14.**		301	9.7625	397.609	190.613	0.9664	13.806	24.161	69.514
**15.**		298	6.5019	164.657	84.551	0.9471	9.195	23.677	68.814
**16.**	**Halsey**	303	0.0806	0.0007	0.0130	0.0066	0.1140	0.1673	5.786
**17.**		301	0.3869	0.0201	0.2994	0.0383	0.5472	0.9576	13.839
**18.**		298	5.937	53.759	70.511	0.8649	8.397	21.622	65.761

Table S5. Thermodynamic parameters for adsorption of SA dye at different temperatures pH 12.01.

Temperature (K)	ΔG° (J/mole)	ΔH° (J/mole)	ΔS°(J/mole)
298	-159.122	75461.909	253.76185
301	-920.407	75461.909	253.76185
303	-1427.93	75461.909	253.76185

Supporting Figures

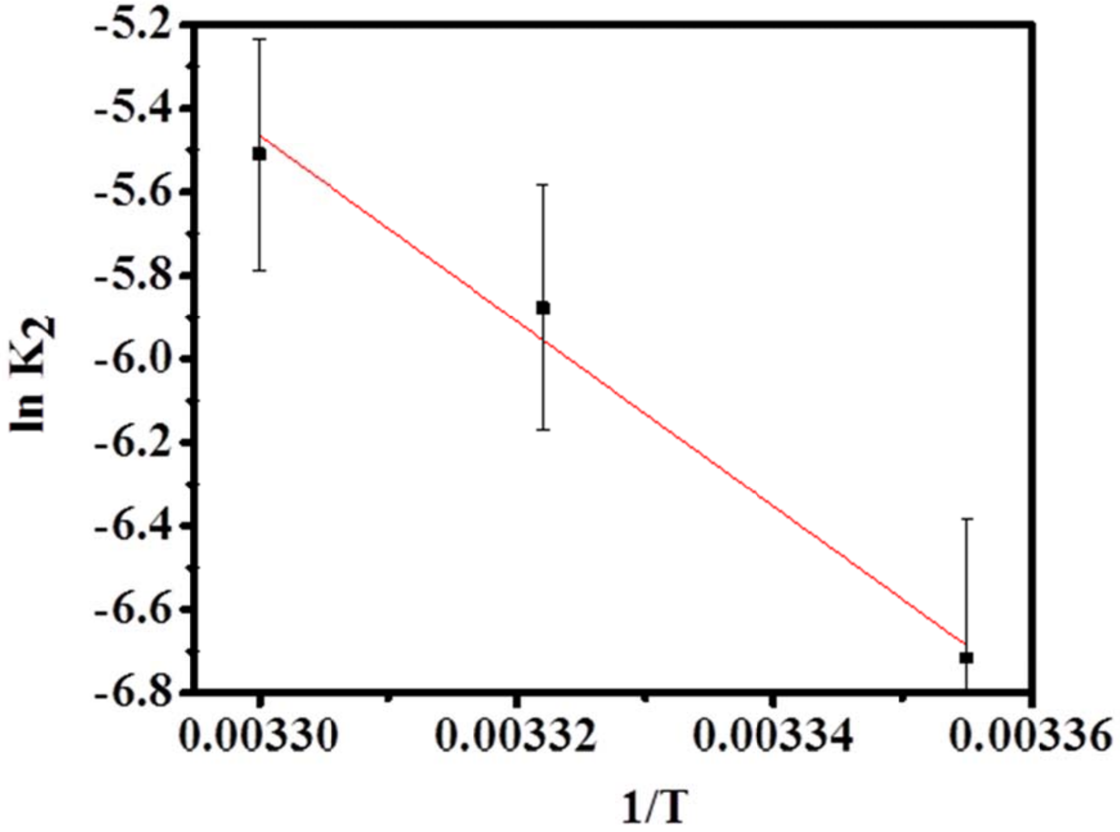

Figure S1. Arrhenius plot.

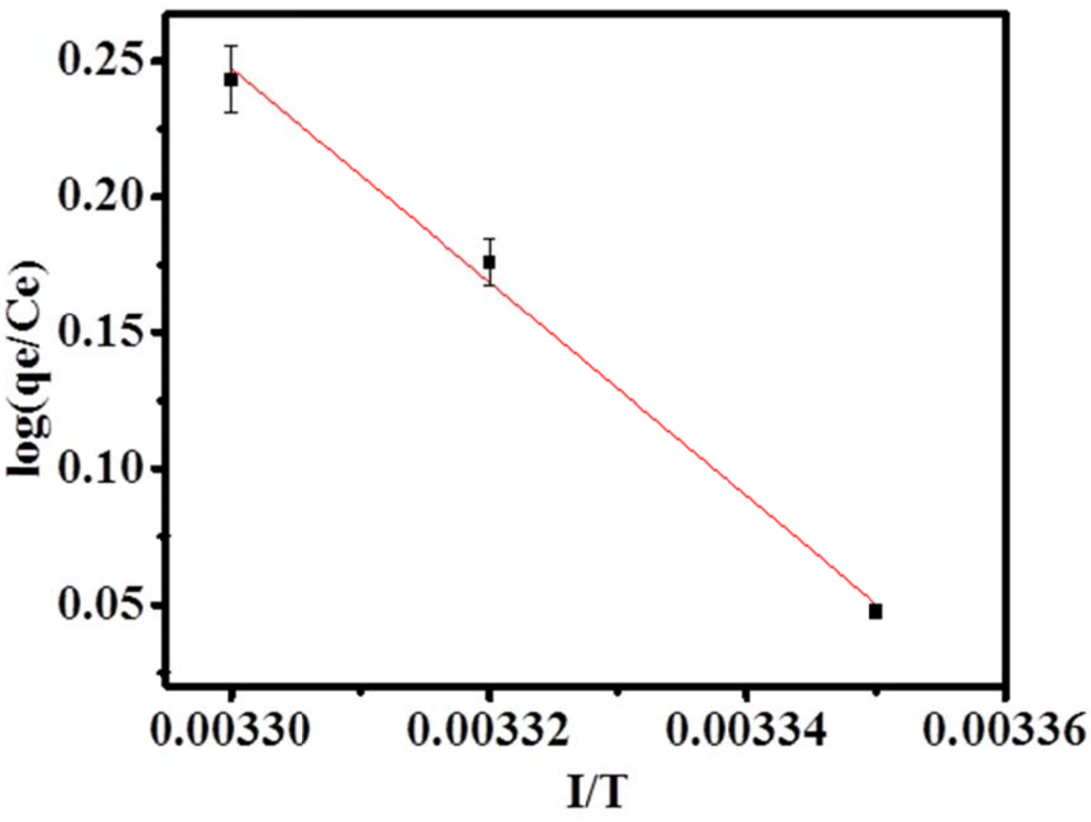

Figure S2. Von’t Hoff plot for adsorption of SA dye.
