# Supplementary figures and images for: Photocatalytic TMO-NMs adsorbent: Temperature-Time dependent Safranine degradation, sorption study validated under optimized effective equilibrium models parameter with standardized statistical analysis
Source: Sci Rep. 2017 Feb 14;7:42509. doi: 10.1038/srep42509 (PMC5307350; doi:10.1038/srep42509)

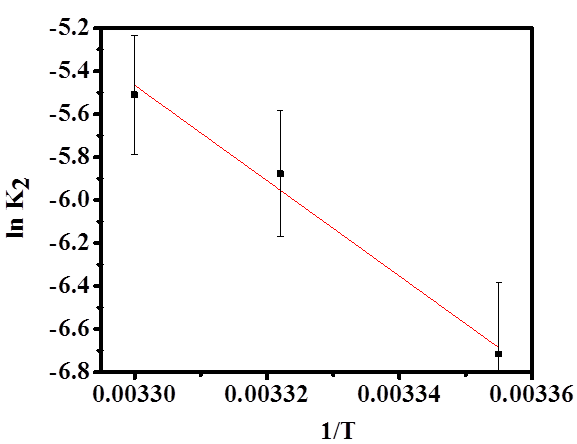

Supplement: Supplementary Figure S1 [file srep42509-s2.tiff]

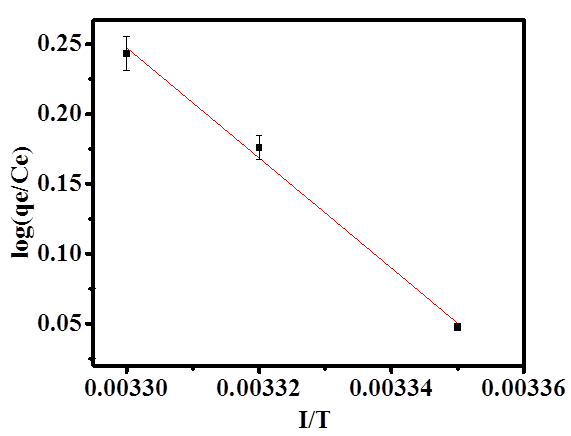

Supplement: Supplementary Figure S2 [file srep42509-s3.tiff]
